# Driving innovations in cancer research through spatial metabolomics: a bibliometric review of trends and hotspot

**DOI:** 10.3389/fimmu.2025.1589943

**Published:** 2025-06-10

**Authors:** Shupeng Chen, Yuzhe Zhang, Xiaojian Li, Ye Zhang, Yingjian Zeng

**Affiliations:** ^1^ School of Clinical Medicine, Jiangxi University of Chinese Medicine, Nanchang, China; ^2^ The First Laboratory of Cancer Institute, The First Hospital of China Medical University, Shenyang, Liaoning, China; ^3^ Hematology Department, Affiliated Hospital of Jiangxi University of Traditional Chinese Medicine, Hematology Department, Nanchang, Jiangxi, China

**Keywords:** spatial metabolomics, immune escape, tumor microenvironment, bibliometric analysis, multi-omics integration, metabolic heterogeneity, research trends, scientific collaboration

## Abstract

**Background:**

Spatial metabolomics has revolutionized cancer research by offering unprecedented insights into the metabolic heterogeneity of the tumor microenvironment (TME). Unlike conventional metabolomics, which lacks spatial resolution, spatial metabolomics enables the visualization of metabolic interactions among cancer cells, stromal components, and immune cells within their native tissue context. Despite its growing significance, a systematic and visualized analysis of spatial metabolomics in cancer research remains lacking, particularly in the integration of multi-omics data and the standardization of methodologies for comprehensive tumor metabolic mapping.

**Objectives:**

This study aims to conduct a bibliometric analysis to systematically evaluate the development trends, key contributors, research hotspots, and future directions of spatial metabolomics in cancer research.

**Methods:**

A bibliometric approach was employed using data retrieved from the Web of Science Core Collection. Analytical tools such as VOSviewer and CiteSpace were utilized to visualize and assess co-citation networks, keyword co-occurrence, and institutional collaborations. Key metrics, including publication trends, authorship influence, country contributions, and journal impact, were analyzed to map the research landscape in this domain.

**Results:**

A total of 182 publications on spatial metabolomics in cancer research were identified over the past two decades, with a notable surge in research output beginning in 2018. The field has experienced accelerated growth, with an annual average of 40 publications since 2021, reflecting its increasing relevance in cancer studies. Among 28 contributing countries, China (n=53), the United States (n=35), Germany (n=18), and the United Kingdom (n=13) have been the most active contributors. China leads in publication volume, while the United States exhibits the highest citation impact, indicating significant research influence. International collaboration networks are particularly strong among the United States, Germany, and China, underscoring the global interest in this emerging field. Analysis of key authors and institutions identifies He Jiuming as the most prolific author and Song Xiaowei as the researcher with the highest average citations. Other influential authors include Abliz Zeper and Sun Chenglong. Leading research institutions driving advancements in this field include the Chinese Academy of Medical Sciences, Peking Union Medical College, Harvard Medical School, and Stanford University. Regarding journal impact, Nature Communications (n=11), Journal of Pharmaceutical Analysis (n=9), and Nature Methods (n=8) are the most active publishing platforms in this domain. Citation analysis reveals that Cell, BioEssays, and Genome Medicine are among the most highly cited journals, reflecting the interdisciplinary nature of spatial metabolomics research.

## 1 Introduction

Cancer remains one of the most severe global public health challenges, with its incidence and mortality rates continuously rising. According to statistics, approximately 20 million new cancer cases were reported worldwide in 2022, with related deaths reaching 9.7 million (1). Despite significant advancements in early cancer screening, precision diagnosis, and targeted therapy, the efficacy of existing treatment strategies remains limited due to the high heterogeneity of cancer and its adaptability to therapeutic interventions (2, 3). Tumor cells can reprogram their metabolic pathways to adapt to changes in the microenvironment, thereby promoting tumorigenesis, progression, and drug resistance (4). Therefore, an in-depth understanding of the spatial distribution characteristics of tumor metabolic reprogramming is crucial for precision medicine and personalized treatment.

In recent years, spatial metabolomics (SM), an emerging omics technology, has enabled *in situ* detection of metabolite spatial distributions in tissue sections and their biological functions through high-resolution imaging mass spectrometry (MSI) (5). Compared to traditional metabolomics methods such as liquid chromatography-mass spectrometry (LC-MS) and gas chromatography-mass spectrometry (GC-MS), spatial metabolomics provides information on metabolic heterogeneity across different tissue structures, overcoming the limitations of metabolic studies at the cellular and tissue levels (6). With technological advancements, various MSI techniques, including matrix-assisted laser desorption/ionization mass spectrometry imaging (MALDI-MSI), desorption electrospray ionization mass spectrometry imaging (DESI-MSI), and secondary ion mass spectrometry imaging (SIMS-MSI), have been widely applied in cancer research. These techniques have uncovered key processes in tumor microenvironment adaptation, immune evasion, and drug resistance.

Notably, spatial metabolomics offers unique opportunities to elucidate the mechanisms of immune evasion and metabolic reprogramming at the tumor–immune interface. By mapping metabolite distributions *in situ*, it enables the characterization of localized nutrient competition—such as glucose and tryptophan depletion—which directly contributes to T cell exhaustion and the activation of immune checkpoints like PD-1/PD-L1 (7, 8). Spatial metabolomics also facilitates the profiling of metabolites secreted by tumor-associated macrophages (TAMs) and myeloid-derived suppressor cells (MDSCs), both of which play central roles in orchestrating immunosuppression. Recent studies have demonstrated that MALDI-based spatial metabolomic imaging can visualize the distribution of metabolites such as glycogen at high resolution, revealing spatial heterogeneity closely linked to tumor type, tissue architecture, and microenvironmental dynamics (9). These insights provide new perspectives on how metabolic reprogramming underpins impaired immune surveillance and resistance to immunotherapy. Therefore, integrating spatial metabolomics into immuno-oncology research holds great potential for identifying novel predictive biomarkers, optimizing immune checkpoint blockade strategies, and improving clinical outcomes in cancer immunotherapy.

Bibliometrics is a discipline that employs mathematical and statistical methods to analyze scientific literature. Its primary objective is to evaluate and quantify the distribution, structure, and growth of scientific publications, as well as their interrelationships, to reveal research trends, hotspots, and interdisciplinary collaborations (10). This approach allows researchers to assess scientific activities and impact within a specific domain, identify key journals and publications, and track research collaboration networks. Moreover, visualization techniques facilitate the graphical representation of complex datasets, making patterns and trends in data more intuitive and comprehensible. Therefore, this study aims to systematically review the research progress in the field of cancer spatial metabolomics through bibliometric analysis and visualization techniques. By deeply exploring and analyzing potential insights in this domain, this work seeks to provide valuable references and guidance for future research endeavors.

## 2 Materials and methods

### 2.1 Data source and literature search

The literature data was obtained from the Web of Science Core Collection (WoSCC) database, and the search time was from 1 January 2000 to December 31, 2023. The search formula used was(TS = (tumor OR tumors OR tumor OR cancers OR cancer OR oncology OR neoplasm OR carcinoma OR arcinoma OR carcinomas OR carcinosis OR “hematologic malignancies” OR “blood cancer” OR “leukemia” OR “lymphoma” OR “multiple myeloma”) AND TS = (“Spatial Metabolomics” OR “spatial-resolved metabolomics” OR “spatially resolved metabolomics” OR “Spatial omics”)).

### 2.2 Data screening

#### 2.2.1 Inclusion criteria

(1) Literature related to phenomics and cancer;(2) Literature published in English; (3) Literature types include clinical trial studies, *in vitro* experimental studies, *in vivo* experimental studies, public database analysis studies, reviews, etc.; (4) Literature with complete bibliographic information(including title, country, author, keywords, source).

#### 2.2.2 Exclusion criteria

(1) Conference papers, newspapers, patents, achievements, health and popular science literature, etc.; (2) Duplicate publications;(3) The literature cannot be fully obtained.

The inclusion and exclusion process is independently conducted by two reviewers. If the inclusion and exclusion results are inconsistent, the third reviewer will participate in the work.

#### 2.2.3 Data standardization

After screening, the literature was exported in Refworks and plain text formats. Special characters and redundant spaces were removed. To ensure consistency and reproducibility in the bibliometric analysis, a structured keyword standardization protocol was implemented. First, all extracted keywords were cleaned to remove typographical inconsistencies, redundant punctuation, and spacing anomalies. Next, synonymous terms were merged based on a combined approach of ontology referencing (including MeSH and UMLS concepts), co-occurrence clustering, and manual curation by domain experts. For example, “spatial metabolomics” and “metabolic imaging” were unified under the term “spatial metabolomics”, while “mass spectrometry imaging” and its abbreviation “MSI” were standardized as “MSI-based metabolomics”. Similarly, disease-related terms such as “lung carcinoma” and “lung adenocarcinoma” were consolidated under “lung cancer metabolomics” to improve thematic coherence. All original terms, standardized forms, and associated merging rationales are provided in **Supplementary Table S1**. The categorization process was independently validated by two reviewers, with disagreements resolved by a third expert to ensure methodological rigor. Country/Region names were standardized for consistency in bibliometric analysis. For example, “Hong Kong”, “Macau”, and “Taiwan” were categorized under “China”, while “Scotland”, “Wales”, and “England” were grouped under “United Kingdom”. Subsequently, the Data Import/Export function in CiteSpace software was used to convert and process the retrieved literature, ensuring the uniformity of metadata for further analysis.

#### 2.2.4 Data analysis

##### 2.2.4.1 Data extraction

The normalized text data were incorporated into structured spreadsheets using Microsoft Excel, following a predefined extraction template developed by two researchers. These researchers independently extracted publication attributes (e.g., year, country, institution, authors, keywords, citations), and discrepancies were identified through cross-check comparison. In cases of disagreement, a third senior researcher adjudicated and finalized the extracted values to ensure consistency and reliability. The extracted data includes the following parts: publication information, encompassing the year of publication, country/region, issuing organization, issuing journal, authors, cited literature, and keywords.

##### 2.2.4.2 Analysis methods

This study utilizes bibliometric visualization analysis to systematically review and uncover latent patterns in the domain of cancer spatial metabolomics. Publication volume trends were extracted from CiteSpace outputs and fitted using polynomial regression models in Excel to forecast future trajectories. For country and region-based analysis, co-authorship and publication frequency data were derived from VOSviewer and visualized using Tableau Public.

Institutional collaboration networks were constructed using VOSviewer’s association strength algorithm and refined via Pajek for network structure optimization. Journal and author impact metrics were analyzed through CSV exports from VOSviewer, applying bibliographic coupling and co-citation analysis techniques.

**Figure 1:**
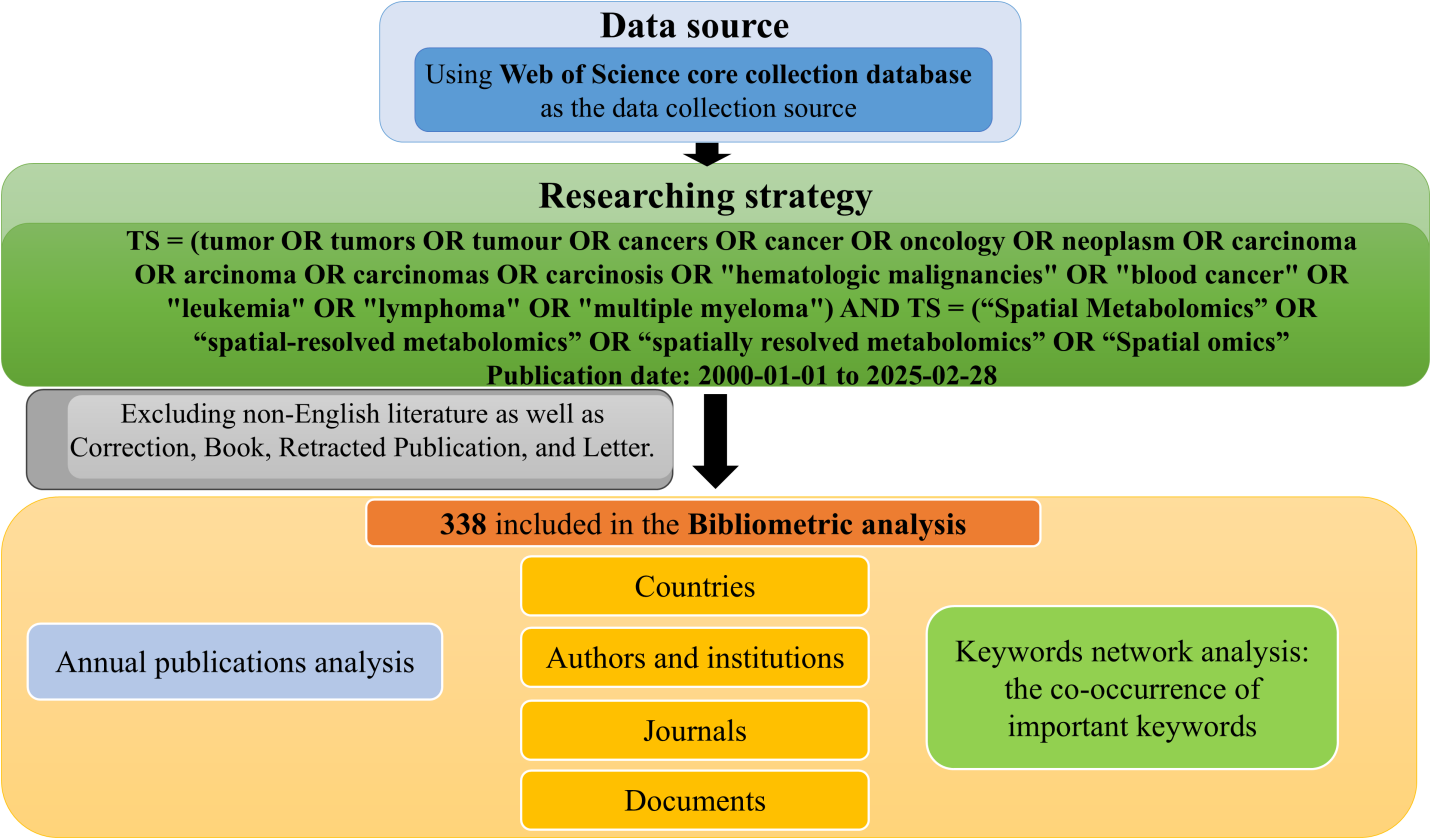
Process of bibliometric analysis of cancer spatial metabolomics.

Keyword co-occurrence and clustering were performed using CiteSpace, where the log-likelihood ratio (LLR) was employed for cluster labeling, and modularity Q and silhouette S values were calculated to assess clustering validity. For thematic visualization, keywords were converted to XML and imported into Carrot² for topic modeling and bubble chart generation. These multi-tool, algorithm-integrated approaches ensured analytical depth and reproducibility across multiple bibliometric dimensions. The data acquisition and analysis workflow is illustrated in **Figure 1**.

## 3 Results

### 3.1 Publication volume

**Figure 2:**
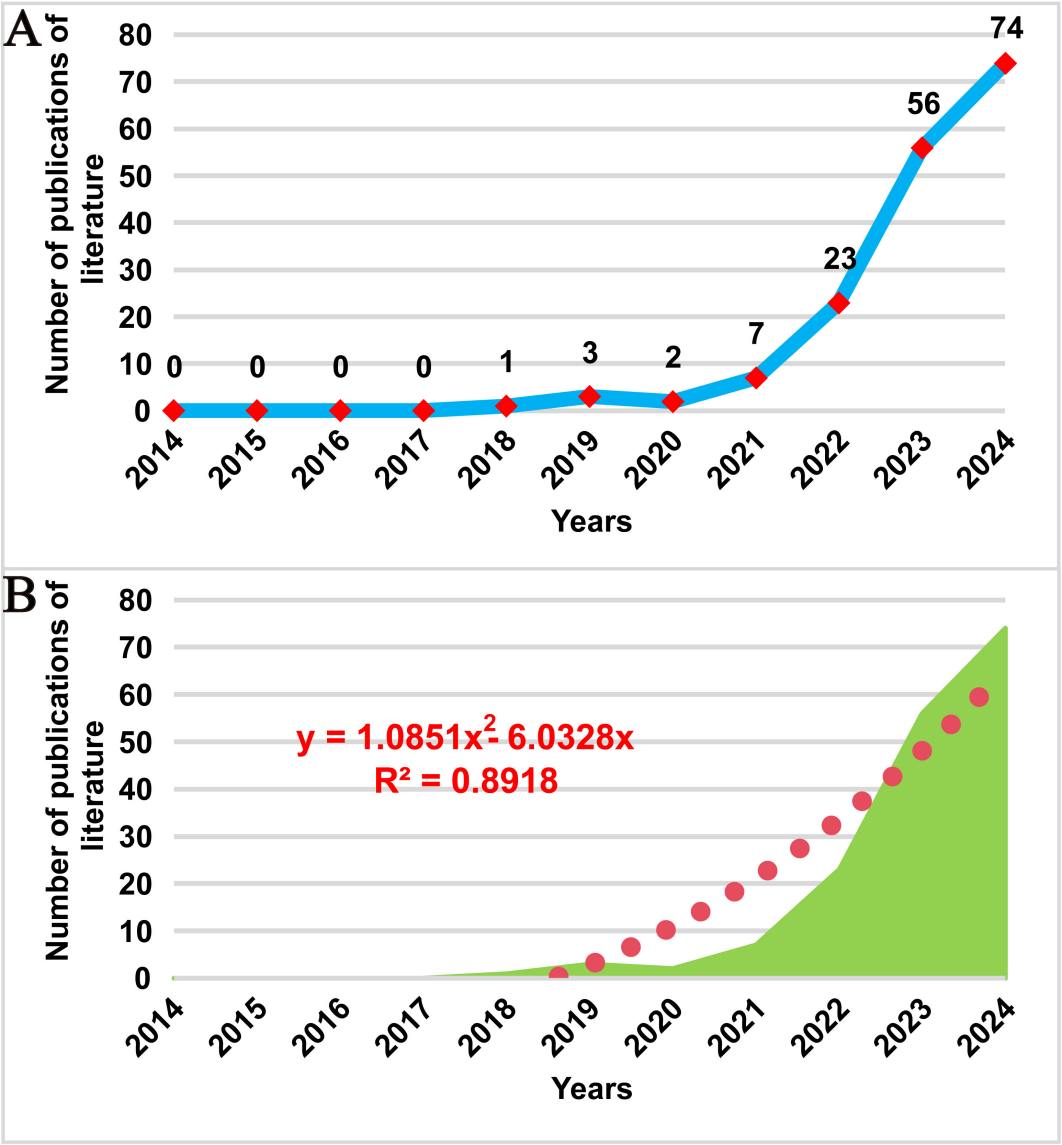
Trend of publications on cancer spatial metabolomics research. **(A)**Trends in the number of publications on cancer spatial metabolomics research from 2014 to 2024; **(B)** Polynomial fitting analysis of the number of publications on cancer spatial metabolomics research from 2014 to 2024.

**Figure 2A** illustrates the trend in publication volume over the past 20 years. The first research on spatial omics in the field of oncology emerged in 2018, marking the beginning of a continuous increase in the number of publications. During the period from 2018 to 2021, the number of publications grew steadily, while from 2021 to 2024, the publication rate accelerated significantly, with an average annual publication volume of 40 papers. To further predict future development trends accurately, a polynomial fitting curve was generated, as shown by the red dashed line in **Figure 2B**. The results indicate that the number of publications in this field will continue to rise. The coefficient of determination (R² = 0.8918) suggests that the model explains 89.18% of the data variability, demonstrating a high reference value for predicting future trends. The analysis of publication volume highlights that spatial metabolomics in oncology is currently a research hotspot and is expected to exhibit promising future growth in this domain.

### 3.2 Countries/regions

**Figure 3:**
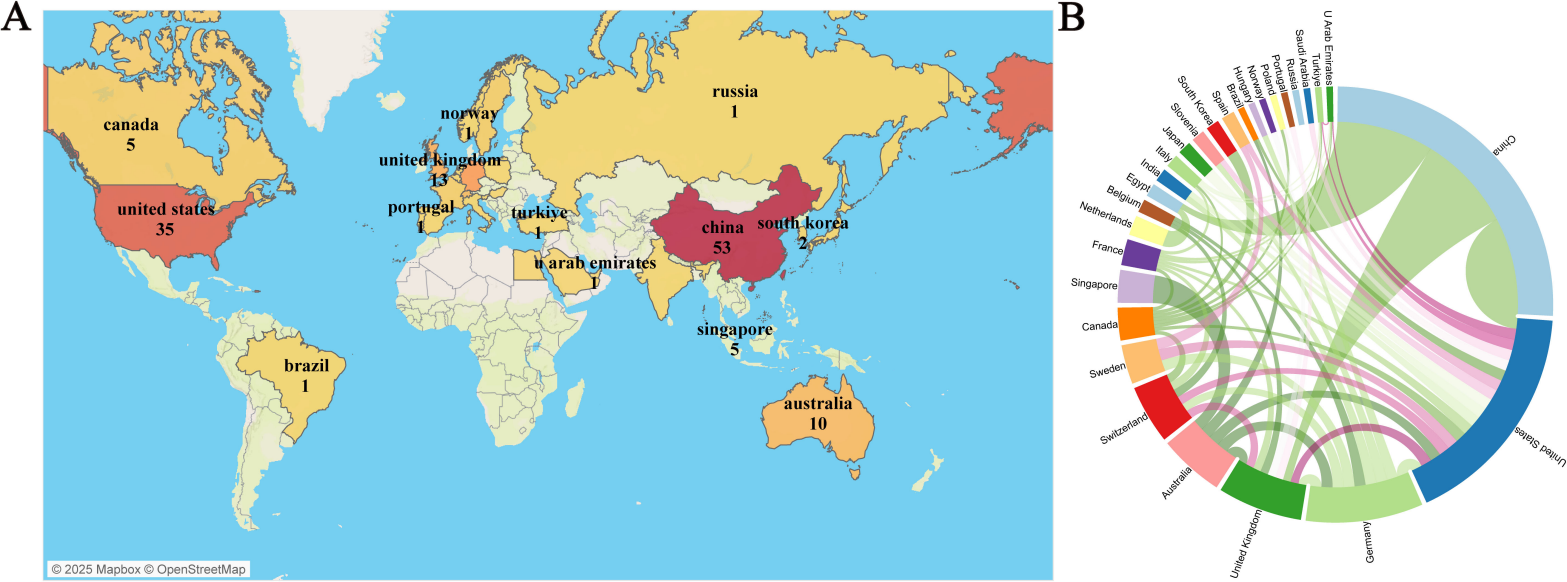
Visualization of Tumor Spatial Metabolomics by Country/Region. **(A)** Global geographic visualization; **(B)** Global cooperation string chart, with nodes representing the number of publications and lines representing the strength of cooperation.

Over the past 20 years, authors from 28 countries/regions have published research in this field. **Figure 3A** presents a geographical visualization of the global literature on spatial metabolomics in oncology. Among these, China has the highest number of publications (n = 53), followed by the United States (n = 35), Germany (n = 18), and the United Kingdom (n = 13). **Figure 3B** illustrates the chord diagram of international collaborations. The United States exhibits the highest cooperation intensity (n = 30), followed by Germany (n = 19). **Table 1** provides a detailed overview of the top 10 publishing countries, including key metrics such as Publication volume, Cooperation intensity, Total citations, and Average citation per paper. Among them, China has the highest total citations (1011), while the United States leads in average citations per paper (47.42), followed by China (39.96), the United Kingdom (25.90), and Germany (21.89). The geographical distribution of publications indicates that research on spatial metabolomics in oncology has garnered worldwide attention and holds significant influence in the scientific community.

**Table 1:** The top 10 countries according to the total publications.

Rank	Country	Publication	Cooperation intensity	Total citations	Average citation
1	China	53	7	1011	39.9608
2	United States	35	30	961	47.4216
3	Germany	18	19	290	21.8864
4	United Kingdom	13	16	643	25.9047
5	Australia	10	8	324	7.9177
6	Switzerland	9	8	111	18.8489
7	Sweden	6	4	495	4.4553
8	Canada	5	9	36	8.5926
9	Singapore	5	1	32	2.6755
10	France	4	7	8	1.9095

### 3.3 Institutions and authors

**Figure 4:**
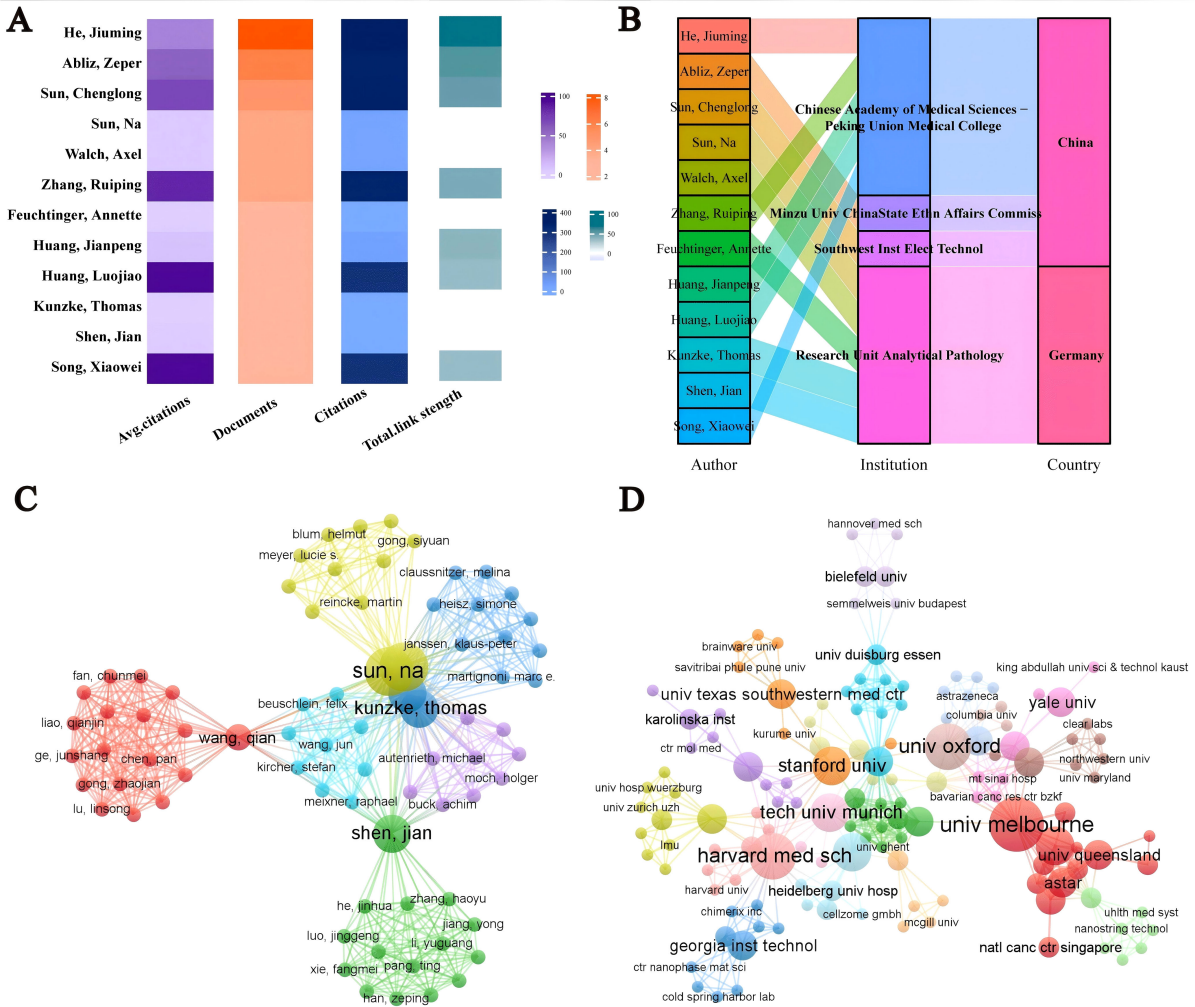
Visualization analysis of authors/institutions in cancer spatial metabolomics research. **(A)** Core authors with over 3 published articles; **(B)** Core Author Institution and Country; **(C)** Author Collaboration Network; **(D)** Agency Cooperation Network.

Over the past 20 years, 947 authors from 142 institutions worldwide have published research on spatial metabolomics in oncology. **Figure 4A** presents the publication data for authors with more than three publications. Song Xiaowei has the highest average citation per paper, while He Jiuming is the most prolific author. He Jiuming, Abliz Zeper, and Sun Chenglong are among the most cited authors. Most of these authors are affiliated with institutions such as the Chinese Academy of Medical Sciences & Peking Union Medical College, Minzu University of China & State Ethnic Affairs Commission, Southwest Institute of Electronic Technology (China), and the Research Unit Analytical Pathology (Germany), as illustrated in **Figure 4B**. **Figure 4C** depicts the author collaboration network, highlighting seven core research teams that are driving advancements in this field, led by Sun Na, Wang Qian, Shen Jian, Beuschlein Felix, Kunzke Thomas, Janssen Klaus-Peter, and Autenrieth Michael. **Figure 4D** illustrates the institutional collaboration network, showing that institutions such as Harvard Medical School, the University of Melbourne, and Stanford University maintain close collaborations with other research institutions, significantly contributing to the development of this discipline.

### 3.4 Journals

**Figure 5:**
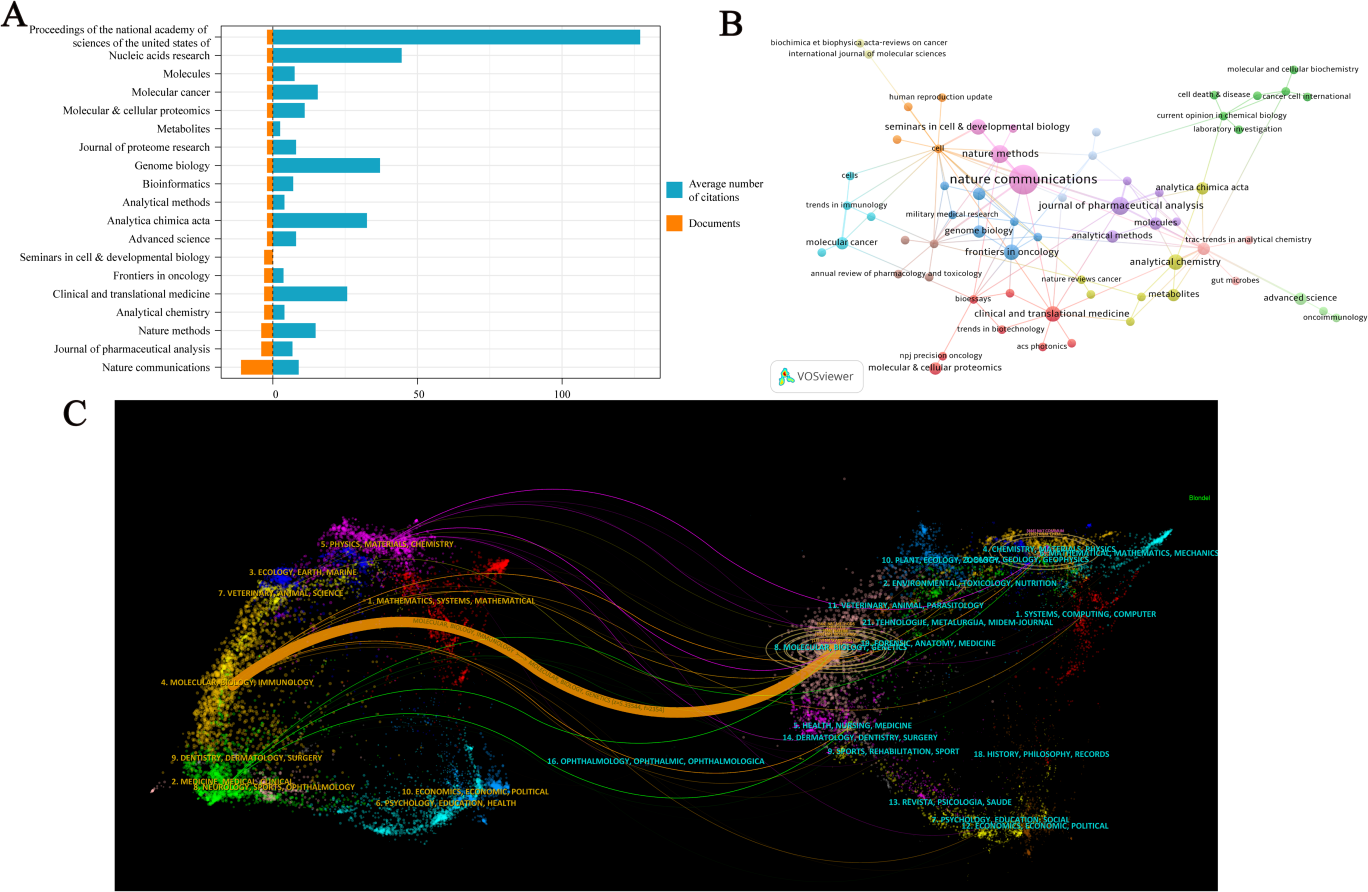
Visualization Analysis of Journals in Cancer Spatial Metabolomics Research. **(A)** Journals with more than 2 articles; **(B)** Journal co citation network graph; **(C)** Journal Double Image Overlay.

A total of 96 journals have published research on spatial metabolomics in oncology. **Figure 5A** presents journals with at least two publications in this field. Among them, Nature Communications (n = 11) has the highest number of publications, followed by the Journal of Pharmaceutical Analysis (n = 9) and Nature Methods (n = 8). The Proceedings of the National Academy of Sciences of the United States of America (PNAS) has the highest average citations per paper (C = 127). **Figure 5B** illustrates the citation network of journals. Cell (C = 433) is the most cited journal, followed by BioEssays (C = 325), Genome Medicine (C = 298), Proceedings of the National Academy of Sciences of the United States of America (C = 254), Journal of Pharmaceutical Analysis (C = 254), and Trends in Immunology (C = 238). The dual-map overlay of journals reveals the citation relationships between citing and cited journals. On the left, the clusters of citing journals represent the knowledge frontiers in this field, while on the right, the clusters of cited journals represent the fundamental knowledge base of the field. As shown in **Figure 5C**, the orange path indicates that journals in Molecular Biology and Genetics are most likely to be cited by journals in Molecular Biology and Immunology, suggesting that recent research includes a significant amount of interdisciplinary work. Meanwhile, the green path shows that research from Molecular Biology, Genetics, Health, Nursing, Medicine, Psychology, Education, and Social Sciences is most likely to be cited by journals related to Medicine, Medical, and Clinical research, implying that this field exhibits strong multidisciplinary integration and convergence.

### 3.5 Keywords

**Figure 6:**
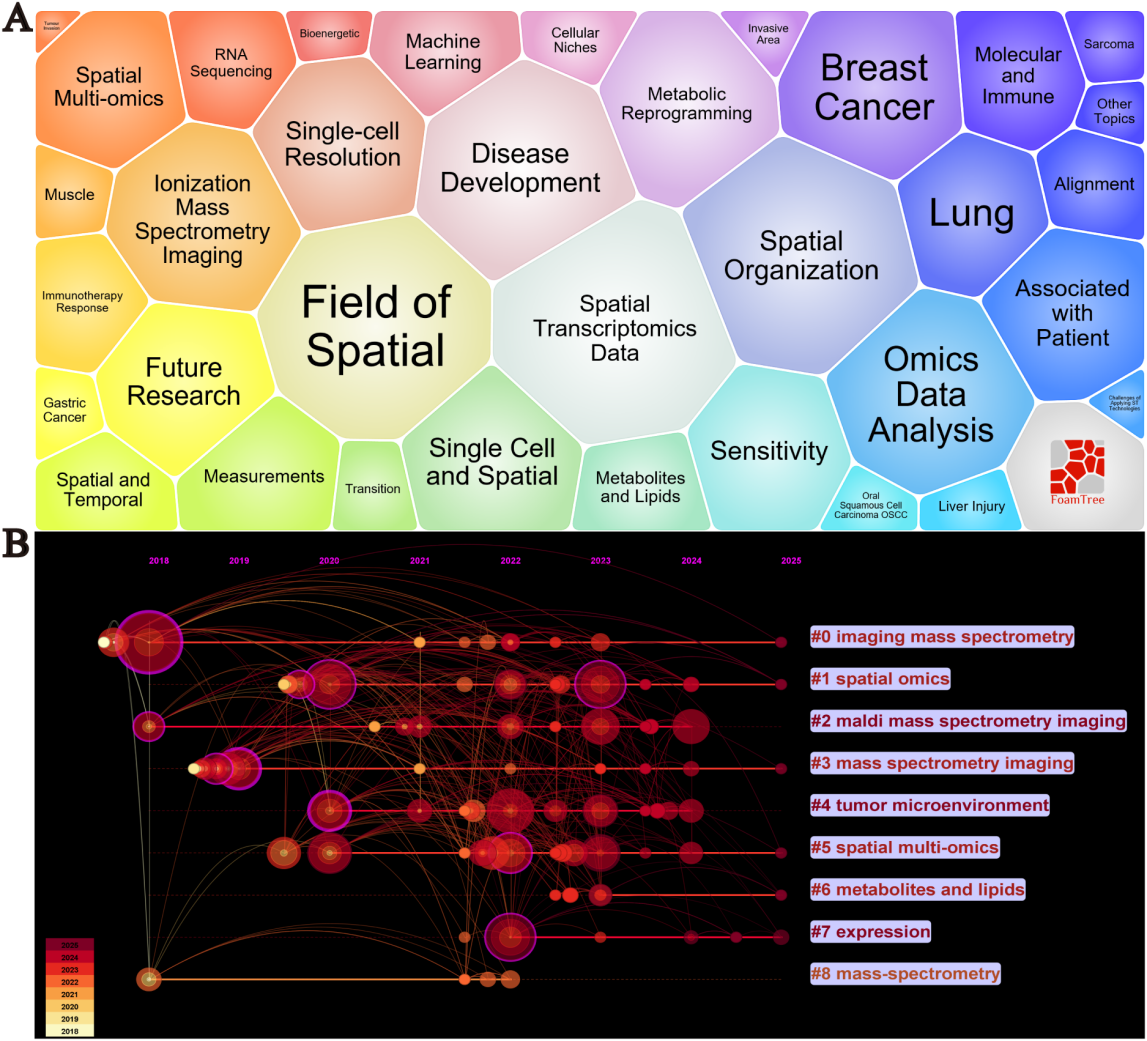
Visualization analysis of keywords in cancer spatial metabolomics research. **(A)** Keyword Bubble Chart, **(B)** Keyword Timeline Clustering Analysis.

By conducting a visualized analysis of keywords from the collected literature, we obtained key insights into the current research landscape. **Figure 6A** shows the bubble plot of keywords, including Spatial Transcriptomics Data Spatial Organization, Metabolic Reprogramming, Molecular and Immune, Disease Development, Single-cell Resolution, Machine Learning, Immunotherapy Response The waiting module is the focus of cancer spatial metabolism research, **Figure 6B** displays a timeline of key developments. Between 2018 and 2025, nine major research clusters have emerged in the field of spatial metabolomics in oncology. Through keyword clustering analysis, we identified three significant research themes that encapsulate both the core content of this field and its transition from fundamental research to applied studies.

These themes are as follows:(1) Spatial Heterogeneity of the Tumor Microenvironment (Core keywords: #4 Tumor Microenvironment, #2 MALDI Mass Spectrometry Imaging, #3 Mass Spectrometry Imaging) The tumor microenvironment (TME) is a complex ecosystem composed of cancer cells, immune cells, stromal cells, and the extracellular matrix. Spatial metabolomics techniques, such as MALDI mass spectrometry imaging (MALDI-MSI) and imaging mass spectrometry, enable precise mapping of metabolite and lipid distributions within tumors. These studies provide insights into intratumoral heterogeneity, offering critical information on tumor invasiveness, drug resistance, and immune evasion mechanisms. (2) Spatial Distribution of Metabolites and Lipids and Their Biological Significance (Core keywords: #6 Metabolites and Lipids, #7 Expression) Understanding the spatial distribution of metabolites and lipids is essential for deciphering tumor metabolic regulation. Imaging mass spectrometry can identify specific metabolite and lipid distribution patterns in tumor tissues, which are closely linked to tumor invasiveness, drug sensitivity, and immune responses. For example, the accumulation of certain metabolites may correlate with hypoxic regions or the formation of immunosuppressive microenvironments, thereby influencing tumor biology and therapeutic response. (3)Integration of Spatial Multi-Omics Technologies (Core keywords: #1 Spatial Omics, #5 Spatial Multi-Omics, #8 Mass-Spectrometry) Spatial multi-omics technologies integrate spatial transcriptomics, proteomics, and metabolomics, offering a comprehensive molecular characterization of tumors. This multi-dimensional integration enables a more systematic understanding of cellular interactions, metabolic regulation, and signaling pathways within the tumor microenvironment. For instance, spatial multi-omics can reveal the spatial organization and function of cancer-associated fibroblasts (CAF) and tumor-associated macrophages (TAM), providing critical insights for identifying new therapeutic targets and developing personalized treatment strategies.

### References

3.6

**Figure 7:**
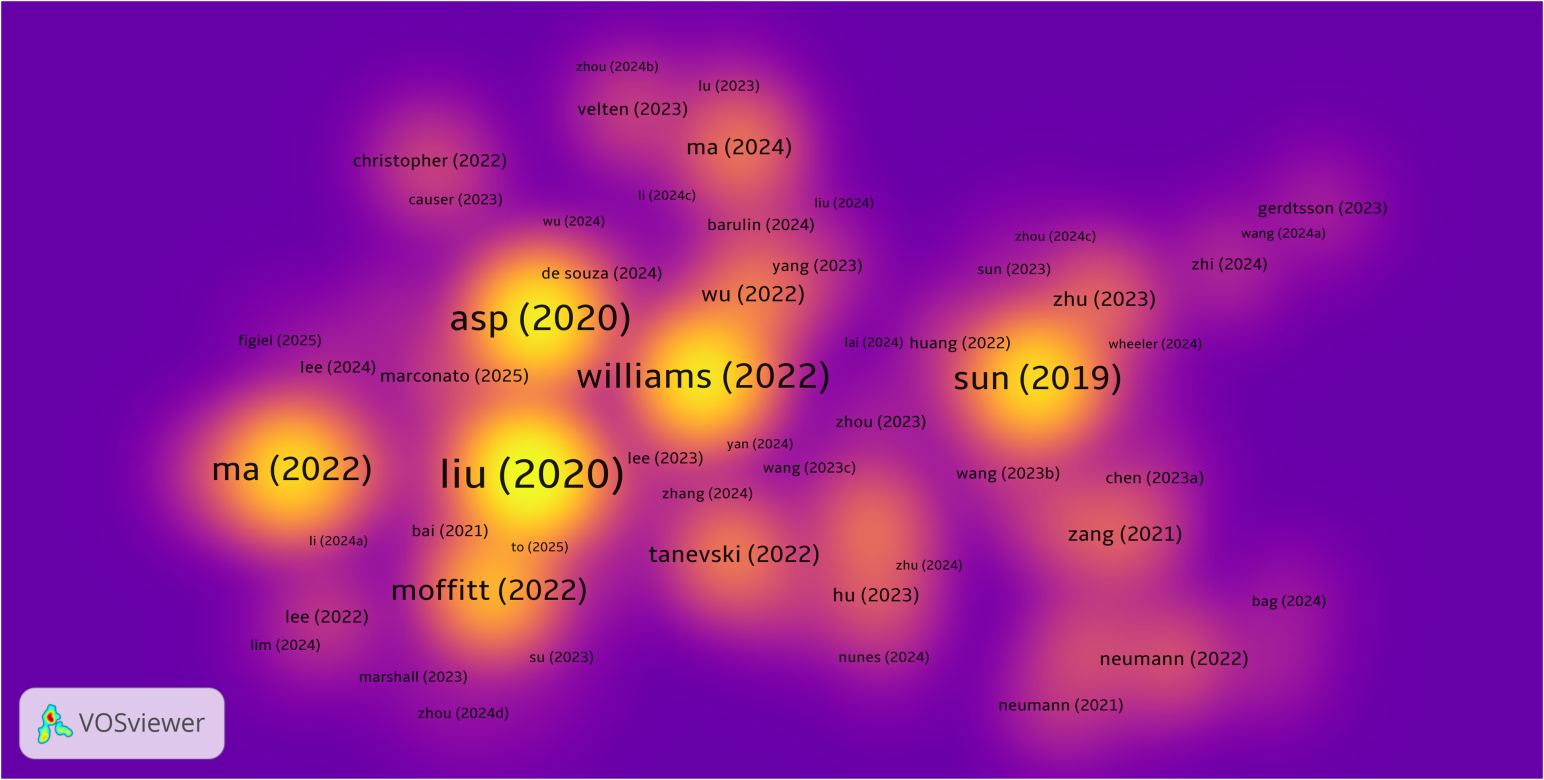
Density map of highly cited literature in cancer spatial metabolomics.

By analyzing highly cited literature, we can further explore research trends and key turning points in the field of spatial metabolomics in cancer. **Figure 7** presents a density map of cited literature, illustrating that the development of cancer spatial metabolomics has progressed from initial exploration to multi-dimensional integration. In 2019, Sun et al. (11) proposed a spatial metabolomics method based on environmental mass spectrometry imaging, which enabled the *in situ* identification of tumor-associated metabolites and metabolic enzymes in tissues. Their study analyzed 256 esophageal cancer tissue samples, constructing spatial distribution maps of metabolites, thereby offering a new perspective on tumor metabolism.

In 2020, Asp (12) systematically categorized spatial transcriptomics technologies, defining five major methodological approaches, which provided technical guidance for subsequent research. In the same year, Liu et al. (13) developed Deterministic Barcoding in Tissue Sequencing (DBiT-seq), a microfluidics-based method that achieved 10 μm resolution in spatial transcriptomics and proteomics sequencing, significantly enhancing the precision of spatial omics research.

In 2022, Black et al. (14) summarized various spatial transcriptomics approaches, including mRNA molecule localization, cell positioning imaging, and mRNA probe spatial arrays, offering important references for experimental design. Moffitt further reviewed the development of spatial genomics, transcriptomics, and proteomics, emphasizing their crucial role in resolving cellular heterogeneity, mapping complex tissue structures, and studying dynamic changes in development and disease processes.

In the same year, Ma et al. (14) reviewed recent advances in mass spectrometry imaging (MSI) applications in oncology over the past five years, covering spatial lipidomics, glycomics, multimodal imaging, and AI/machine learning applications in MSI data analysis. Their work highlighted the extensive potential of spatial metabolomics in cancer research.

To further extract valuable insights, we compiled a list of the top 20 most cited publications, as shown in **Table 2**. Overall, the research trajectory of spatial metabolomics in oncology has evolved from initial applications in 2019, through technological classification and novel methodologies in 2020, to multi-layered spatial omics analyses in 2022. This progression demonstrates a deepening and diversification of the field, providing new tools and perspectives for unraveling tumor metabolism and its microenvironment.

**Table 2:** Highly Cited Literature on Cancer Spatial Metabolomics Research (Top 20).

No.	Author(s)	Journal	Year	Citations	Key Findings
1	Yang Liu et al (13)	Cell	2020	433	Developed DBiT-seq for spatial omics sequencing, achieving 10 μm resolution for transcriptomics and proteomics.
2	Michaela Asp et al (12)	BioEssays	2020	325	Classified spatial transcriptomics into five techniques, including *in situ* sequencing, spatial capture, and imaging.
3	Cameron G. Williams et al (15)	Genome Medicine	2022	298	Reviewed different spatial transcriptomics methods and their applications in RNA spatial mapping.
4	Chenglong Sun et al (11)	PNAS	2019	253	Introduced spatially resolved metabolomics using ambient MSI to map tumor-associated metabolites in esophageal cancer.
5	Joshua R. Moffitt et al (16)	Nature Reviews Genetics	2022	160	Discussed the role of spatial omics in cellular heterogeneity, tissue structure, and disease dynamics.
6	Chang Xu et al (17)	Nucleic Acids Research	2022	84	Developed DeepST, a deep learning framework for detecting spatial domains in spatial transcriptomics.
7	Delphine Parrot et al (18)	Planta Medica	2018	78	Reviewed DESI-IMS as a tool for spatial metabolomics, providing molecular insights into biological systems.
8	Jovan Tanevski et al (19)	Genome Biology	2022	72	Proposed MISTy, a machine learning framework for analyzing highly multiplexed spatial omics data.
9	Yingcheng Wu et al (20)	Clinical and Translational Medicine	2022	68	Summarized advances in spatial omics for tumor microenvironment profiling using high-throughput techniques.
10	Xin Ma et al (21)	Mass Spectrometry Reviews	2024	65	Reviewed MSI applications in cancer, covering spatial lipidomics, glycomics, and multimodal imaging.
11	Wan-Chen Hsieh et al (22)	Journal of Biomedical Science	2022	56	Investigated spatial multi-omics in analyzing the tumor immune microenvironment (TIME).
12	Zhuxian Zhu et al (23)	Gut Microbes	2023	47	Showed Akkermansia muciniphila migrates to lung cancer tissues, altering metabolism and the microenvironment.
13	Qingce Zang et al (24)	Analytica Chimica Acta	2021	39	Developed high-resolution spatial metabolomics (MALDI-MSI) for esophageal cancer with 12 μm resolution.
14	Matthew J. Mosquera et al (25)	Advanced Materials	2022	36	Integrated proteomics, RNA-seq, and spatial omics to study ECM dynamics in prostate cancer.
15	Thomas Hu et al (26)	Nature Communications	2023	35	Proposed single-cell spatial metabolomics (scSpaMet) to analyze protein-metabolite interactions in human tissues.
16	Judith M. Neumann et al (27)	Journal of Cancer Research and Clinical Oncology	2022	32	Used MALDI-MSI to differentiate adenocarcinoma and squamous cell carcinoma in NSCLC with 95% accuracy.
17	Mélanie Planque et al (28)	Current Opinion in Chemical Biology	2023	27	Highlighted MSI (MALDI/DESI) in cancer metabolomics, enabling single-cell resolution mapping of metabolites.
18	Moumita Kundu et al (29)	Molecular Cancer	2024	23	Discussed high-throughput spatial omics for identifying tumor-immune regulatory genes affecting immunotherapy response.
19	Luca Marconato et al (30)	Nature Methods	2025	23	Introduced SpatialData, a computational framework for spatial omics data integration.
20	Renumathy Dhanasekaran et al (31)	Hepatology	2023	22	Reviewed multimodal, single-cell, and spatial omics for characterizing tumor heterogeneity and immune microenvironment.

## 4 Discussion

### 4.1 Research hotspots

#### 4.1.1 Spatial heterogeneity of the tumor microenvironment

The tumor microenvironment (TME) is a highly dynamic and complex ecosystem composed of cancer cells, immune cells, stromal components, and the extracellular matrix (ECM). Its spatial heterogeneity plays a critical role in tumor initiation, progression, and therapeutic resistance.

Wang et al. (32) employed high-resolution MALDI-FT-ICR mass spectrometry imaging to characterize metabolic heterogeneity in adrenocortical carcinoma (ACC). They identified 12 distinct metabolic subregions within tumors using unsupervised clustering and diversity index analysis. Greater intratumoral metabolic heterogeneity was significantly associated with advanced ENSAT stages and poor prognosis. Notably, pathways such as the pentose phosphate and purine metabolism were enriched in highly heterogeneous regions, implicating localized metabolic activity in tumor aggressiveness and treatment resistance.

Chen et al. (33) investigated spatial and genomic heterogeneity in advanced prostate cancer using dual-tracer PET/CT imaging (^18F-DCFPyL and ^18F-FDG) combined with next-generation sequencing. Lesions with low PSMA uptake but high FDG avidity (DCFPyL–FDG+) were predominantly observed in patients with castration-resistant prostate cancer (CRPC) and were associated with visceral metastases, poor PSA response, and unfavorable outcomes. These lesions frequently harbored TP53 and/or RB1 mutations, which were identified as independent risk factors. This study highlights the value of integrated spatial imaging and genomic profiling in delineating tumor heterogeneity and guiding personalized therapy.

Bian et al. (3) developed SplitFusion, a clinically validated fusion detection algorithm optimized for FFPE tumor samples. Demonstrating superior sensitivity and specificity, SplitFusion successfully identified both known and novel gene fusions across RNA sequencing platforms. It also revealed coexisting subclonal fusion variants—such as EML4::ALK v3—in single tumors, unveiling a new dimension of fusion-driven intratumoral heterogeneity. These findings underscore the utility of combining transcriptomics and bioinformatics to advance molecular diagnostics in cancer.

In parallel, advancements in spatial metabolomics—particularly high-resolution ultra-high-performance liquid chromatography-tandem mass spectrometry (HR-UHPLC-MS/MS)—have greatly enhanced our ability to resolve tumor metabolic heterogeneity. These technologies enable precise spatial localization of key metabolites and deepen our understanding of tumor metabolic adaptability (5).

Collectively, these studies highlight the emerging roles of spatial metabolomics, transcriptomics, and multi-modal imaging in unraveling tumor heterogeneity. They not only broaden our mechanistic insights into the TME but also open new avenues for precision diagnostics and personalized therapeutic strategies in oncology.

#### 4.1.2 Spatial distribution of metabolites and lipids and their biological significance

The spatial distribution of metabolites and lipids plays a pivotal role in cancer metabolic adaptation, immune evasion, and therapeutic resistance.

Zhu et al. (34) applied matrix-assisted laser desorption/ionization mass spectrometry imaging (MALDI-MSI) to investigate curcumin-induced metabolic reprogramming in three-dimensional breast cancer tumor spheroids. Their spatial metabolomic profiling revealed that curcumin modulates the abundance and localization of key lipid species—including phosphatidylcholine, phosphatidylethanolamine, and fatty acids—as well as polyamine-related metabolites such as glutamine and spermidine. Notably, curcumin treatment suppressed the expression of lipid and polyamine biosynthetic enzymes (e.g., FASN, SCD, GLS), indicating a broad reconfiguration of tumor metabolic networks. These findings underscore the utility of spatial metabolomics in revealing localized drug responses and highlight curcumin’s potential in modulating cancer metabolism.

Sun et al. (35) conducted a comprehensive spatial metabolomic analysis across multiple organs—including liver, skeletal muscle, visceral and subcutaneous adipose tissue, and serum—in patients with cancer cachexia (CCx). They observed increased metabolic activity in adipose tissues and liver, accompanied by metabolic suppression in skeletal muscle and serum. Energy charge analysis revealed a decline in muscle bioenergetic capacity, in contrast to elevated energy status in liver and adipose compartments. Pathway enrichment and correlation network analyses further demonstrated extensive inter-organ metabolic cross-talk, with the liver serving as a central hub through lipid, amino acid, carbohydrate, and vitamin metabolism. These results highlight the systemic nature of CCx and the critical regulatory role of the liver–adipose–muscle axis in metabolic reprogramming.

In the context of drug resistance and immune modulation, recent studies have uncovered direct links between metabolic remodeling and immune checkpoint regulation. Zou et al. (36) reported that the accumulation of lactic acid in the tumor microenvironment caused by metabolic stress can activate HIF-1α, thereby upregulating PD-L1 expression and promoting immunosuppression. Lactic acid can also promote M2 polarization and regulate the tumor microenvironment by secreting cytokines such as TGF-β and IL-10. Meanwhile, IL-10 can induce the upregulation of PD-L1 expression on monocytes, thereby weakening CD8+ T cell-mediated immune surveillance. Under hypoxic conditions, the activation of HIF-1α can inhibit the activity of α-KGDH, reduce the oxidation of α-ketoglutarate, and lead to the accumulation of succinate. In the tumor microenvironment, succinate can promote the polarization of TAMs to a pro-tumor phenotype through the SUCNR1/PI3K/HIF-1α signaling pathway, induce the secretion of related cytokines by M1 and M2 macrophages, and thereby affect the expression of PD-L1 (37). Similarly, Ma et al. (38) demonstrated that cholesterol-rich membrane domains can stabilize PD-L1 on tumor cells, activate the PI3K/AKT/mTOR signaling pathway, and promote the expression and stability of downstream target protein HIF-1α through this pathway, thereby inhibiting CD8^+^ T cell activity and promoting immune escape.

**Figure 8:**
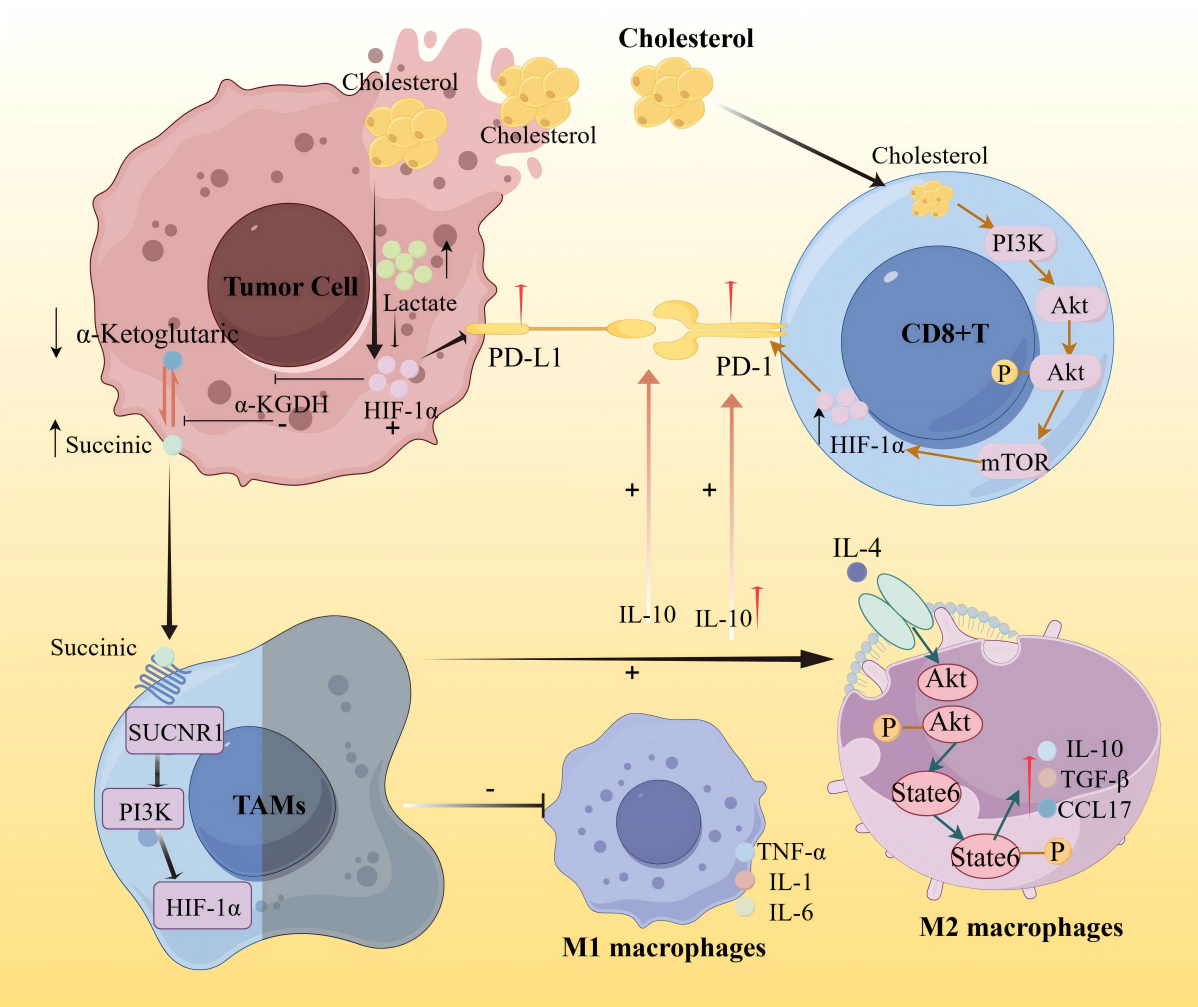
Schematic diagram of the mechanism of lipid metabolism immune interaction promoting immune escape in tumor microenvironment.

Overall, these findings provide mechanistic insights into how metabolic remodeling supports treatment resistance and immune evasion in a spatially restricted manner. A conceptual overview is shown in **Figure 8**, depicting the interactions between lactic acid, cholesterol, α-ketoglutarate, succinate, and key immune effectors including PD1, PD-L1, tumor-associated macrophages (TAMs), and CD8^+^ T cells.

Ongoing advancements in spatial metabolomic technologies—such as MALDI-MSI, SIMS-MSI, and high-resolution mass spectrometry (HR-MS)—are enabling more precise visualization of metabolic-immune interactions at subcellular resolution. Future integration of spatial metabolomics with transcriptomics, proteomics, and immunomics is expected to deepen our understanding of tumor microenvironmental dynamics and inform the development of personalized therapeutic strategies.

This figure illustrates the regulatory mechanism of cholesterol on tumor immune metabolism. On one hand, it activates hypoxia-inducible factor-1α (HIF-1α), thereby transcriptionally up-regulating the expression of programmed death ligand 1 (PD-L1) on tumor cells, promoting immune escape of tumor cells. On the other hand, the increase of cholesterol can activate the PI3K/AKT/mTOR signaling pathway, thereby promoting the expression and stability of downstream target protein HIF-1α, leading to enhanced membrane stability of PD-L1. The continuous expression of PD-L1 further inhibits the activity of CD8^+^ T cells and promotes immune escape. HIF-1α in tumor cells further inhibits the activity of α-KGDH, reduces the oxidation of α-ketoglutarate, and leads to the accumulation of succinate. Succinate promotes the polarization of TAMs to the M2 phenotype through the SUCNR1/PI3K/HIF-1α signaling pathway. M1-type macrophages in the tumor microenvironment can activate immune cells by secreting pro-inflammatory factors (TNF-a, IL-1, IL-6), enhancing anti-tumor immunity. IL-4 can activate M2-type macrophages, secrete IL-10, TGF-β, and CCL17, thereby inhibiting T cell-mediated anti-tumor immune responses. IL-10, on the one hand, can enhance the maintenance of the PD-L1/PD-1 axis, thereby promoting immune escape of tumor cells, and on the other hand, IL-10 induces up-regulation of PD-L1 expression in monocytes, thereby weakening CD8^+^ T cell-mediated immune surveillance.

#### 4.1.3 Integration of spatial multi-omics analysis

The rapid evolution of multi-omics technologies has positioned spatially resolved integration of metabolomics, transcriptomics, proteomics, and other omics layers as a powerful strategy for elucidating tumor metabolic heterogeneity and the dynamic remodeling of the tumor microenvironment (TME). Compared with single-omics approaches, spatial multi-omics enables concurrent mapping of metabolite distribution, gene expression, and protein activity at both cellular and tissue levels, providing a comprehensive view of metabolic reprogramming, immune–metabolic crosstalk, and mechanisms of drug resistance.

Sun et al. (39) conducted a spatial multi-omics analysis in gastric cancer by integrating AFADESI-MSI–based metabolomics, MALDI-MSI–based lipidomics, and 10× Genomics Visium–based spatial transcriptomics. Their study identified distinct metabolic and transcriptional programs across tumor, epithelial, intestinal metaplasia, and lymphoid regions. Of particular interest was the discovery of an immunologically active tumor–normal interface enriched in plasma B cells and Th2-like CD4^+^ T cells, characterized by specific immunometabolic signatures. Spatial co-localization of polyamines, fatty acids, and key metabolic genes such as FASN and GLS suggested coordinated metabolic reprogramming that may drive tumor progression and immune modulation.

Liang et al. (40) systematically reviewed recent applications of spatial multi-omics in gastrointestinal malignancies, including esophageal, gastric, and colorectal cancers. They emphasized that integrating spatial transcriptomic, proteomic, and metabolomic data allows for high-resolution profiling of tumor heterogeneity, cellular interactions, and microenvironmental remodeling. The review highlighted the translational relevance of spatial multi-omics in identifying therapeutic targets, stratifying patients, and informing precision oncology strategies.

Lu et al. (41) employed a spatial multi-omics approach to investigate the metabolic effects of Anlotinib in hepatocellular carcinoma. By integrating metabolomics, transcriptomics, and proteomics, they identified four key metabolic pathways modulated by Anlotinib—steroid biosynthesis, the pentose phosphate pathway, taurine and hypotaurine metabolism, and lipid biosynthesis. Spatial analysis revealed increased abundance and altered localization of metabolites such as 7-ketocholesterol, phosphoenolpyruvate, taurine derivatives, and trans-crotonyl-CoA, which correlated with enhanced CD8^+^ T cell infiltration and reprogramming of the tumor immunometabolic landscape. These metabolites were proposed as potential biomarkers, supporting the clinical promise of Anlotinib as a modulator of both tumor metabolism and immune response.

Despite these advancements, cross-platform integration remains technically challenging due to inconsistencies in spatial resolution, signal dynamics, and detection principles among different technologies. For example, aligning MALDI-MSI or SIMS-MSI metabolomic data with spatial transcriptomics from platforms such as 10x Genomics Visium may introduce batch effects that confound biological interpretation.

To address these challenges, several data harmonization strategies have been developed. ComBat, an empirical Bayes-based method, has proven effective in mitigating batch effects across omics datasets and can be adapted to spatial data. Additionally, Seurat’s integration pipelines—based on canonical correlation analysis (CCA) and mutual nearest neighbor (MNN) algorithms—facilitate robust alignment of transcriptomic and proteomic layers with metabolic features. Furthermore, frameworks such as SpatialData offer standardized spatial metadata management and coordinate referencing, improving reproducibility and enabling cross-study comparability.

In summary, the integration of spatial metabolomics with other omics modalities offers a powerful approach for characterizing cancer metabolic adaptation and microenvironmental complexity. The implementation of robust normalization algorithms and standardized spatial frameworks will be critical for improving reproducibility, validating spatial biomarkers, and advancing therapeutic innovations in metabolic targeting, immunotherapy, and personalized medicine.

### 4.2 Research trends

#### 4.2.1 Deep integration of computational tools and artificial intelligence

Advancements in computational tools and artificial intelligence (AI) are revolutionizing cancer spatial metabolomics by enabling precise analysis of metabolic heterogeneity within the tumor microenvironment (TME) and among diverse interacting cell populations.

Hu et al. (26) introduced scSpaMet, a multi-modal framework that integrates untargeted spatial metabolomics via time-of-flight secondary ion mass spectrometry (TOF-SIMS) with multiplexed protein imaging through imaging mass cytometry (IMC). This approach enables joint spatial profiling of over 200 metabolites and 25 proteins at single-cell resolution across a variety of human tissues. By applying cross-modality image registration and segmentation, scSpaMet identifies metabolically distinct cellular phenotypes and their associated proteomic signatures. Furthermore, the use of deep learning to embed metabolic features into latent spaces allows the inference of metabolic trajectories and the mapping of metabolite–protein interactions and competitive dynamics within the TME, offering valuable insights into tumor evolution and functional heterogeneity.

Ma and Fernández (21) provided a comprehensive review of recent innovations in mass spectrometry imaging (MSI) technologies such as MALDI, DESI, and SIMS highlighting their growing application in spatial cancer metabolomics. The authors emphasized the increasing role of AI and machine learning in enhancing MSI workflows, including feature extraction, dimensionality reduction, unsupervised tissue segmentation, and biomarker discovery. These computational approaches are essential for managing high-dimensional spatial datasets and improving molecular annotation and spatial resolution in oncologic studies.

More broadly, recent advances in spatial metabolomics have underscored the transformative potential of AI for integrating and analyzing large-scale spatial data. By leveraging machine learning algorithms, researchers have significantly improved metabolite identification, enhanced spatial resolution, and enabled cross-omics integration—advancing the interpretation of spatial metabolic networks within both cancer systems biology and metabolic engineering contexts (42).

Despite these achievements, limitations in spatial resolution persist. For instance, MALDI-MSI typically achieves resolutions in the range of 10–20 μm, which may obscure subcellular features and local metabolic gradients around immune cells, thereby limiting the granularity of microenvironmental insights.

To overcome these challenges, emerging platforms such as nano-DESI and AFM-IR now offer submicron spatial resolution. When coupled with AI-powered image analysis and multimodal data integration, these technologies promise to unlock detailed subcellular maps of metabolic activity and improve the precision of spatial interpretation.

In summary, the convergence of spatial metabolomics and artificial intelligence has expanded the analytical toolkit for dissecting metabolic reprogramming and immune regulation with high spatial fidelity. This synergy is accelerating progress in precision oncology and the development of spatially informed therapeutic strategies.

#### 4.2.2 Integration of spatial metabolomics with clinical translation

The rapid advancement of spatial metabolomics has significantly enhanced its translational potential in precision oncology, particularly in cancer diagnosis, treatment monitoring, and the investigation of drug resistance mechanisms.

Shen et al. (43) utilized matrix-assisted laser desorption/ionization mass spectrometry imaging (MALDI-MSI) to analyze neoadjuvant chemotherapy (NAC) responses in non-small cell lung cancer (NSCLC). By profiling spatially resolved tumor metabolites, they constructed a predictive classifier that achieved 81.6% accuracy in identifying NAC responders, outperforming conventional pathological evaluation (62.5%) and TNM staging (54.1%).

While these results underscore the value of spatial metabolic signatures as predictive biomarkers, it is important to note that the model was validated only within a single-institution cohort. Lack of external and prospective validation may limit its generalizability, and multi-center studies are needed to confirm its robustness and clinical applicability.

Zhi et al. (44) integrated spatial transcriptomics and spatial metabolomics to study the malignant transformation from oral submucous fibrosis (OSF) to oral squamous cell carcinoma (OSCC). Their work revealed partial epithelial-mesenchymal transition (pEMT) accompanied by polyamine metabolic reprogramming, showing that OSF-derived OSCC cells could adopt a fibroblast-like phenotype. This transformation contributes to extracellular matrix remodeling and immune evasion, providing mechanistic insights relevant to early detection and preventive strategies in oral cancer.

Furthermore, Xu et al. (45) reviewed the application of spatial metabolomics in addressing cancer drug resistance, highlighting how tumor cells undergo metabolic reprogramming—including enhanced glycolysis, altered amino acid and lipid metabolism, and immunosuppressive metabolic shifts—to adapt under therapeutic pressure. Spatial metabolomics enables the characterization of such resistance-associated metabolic heterogeneity at both the tissue and single-cell levels. The authors emphasize that spatially resolved metabolic profiling holds great promise in uncovering mechanisms of resistance, identifying actionable metabolic targets, and guiding personalized therapeutic strategies.

In conclusion, the integration of spatial metabolomics into translational cancer research is accelerating its application in clinical contexts, offering novel avenues for biomarker discovery, therapeutic monitoring, and individualized treatment of drug-resistant tumors.

### 4.3 Limitations

Despite systematically revealing the development trends and research hotspots of spatial metabolomics in oncology through bibliometric analysis, this study has certain limitations that should be considered. These limitations primarily stem from database coverage constraints and methodological limitations in bibliometric analysis.

First, the data for this study were primarily obtained from the Web of Science Core Collection (WOSCC). While WOSCC covers a large number of high-impact journals and is widely used in bibliometric research, its coverage and data acquisition methods may impact the comprehensiveness of the analysis. WOSCC is biased toward fundamental research and high-impact journals, potentially excluding relevant literature from other databases, such as PubMed, Scopus, Embase, Google Scholar, and IEEE Xplore. This may be particularly relevant for clinical studies, applied research, interdisciplinary research, and emerging fields that might not be fully represented in WOSCC.

Additionally, some non-English publications may not be included in the dataset. Given that spatial metabolomics research has been rapidly advancing in non-English-speaking countries such as China, Japan, and Germany, this limitation may introduce geographical bias, potentially affecting the accuracy of global research hotspot assessments.

## 5 Conclusion

This study presents a comprehensive bibliometric review of spatial metabolomics in oncology, outlining its developmental trajectory, major contributing countries and institutions, and core research themes. Since 2018, the field has experienced rapid growth, with increasing global engagement led by China, the United States, Germany, and the United Kingdom. Prominent institutions such as the Chinese Academy of Medical Sciences, Harvard University, and Stanford University have played pivotal roles, while leading journals like Nature Communications and Nature Methods have served as primary platforms for scholarly dissemination.

The primary research focuses in this domain include: (1) spatial heterogeneity of the tumor microenvironment, (2) spatial distribution of metabolites and lipids, and (3) integration of spatial multi-omics. Continued advancement will depend on incorporating high-resolution imaging, single-cell metabolomics, and AI-driven analytics to improve tumor metabolic profiling and support precision oncology.

To propel the field forward, we offer several actionable recommendations. First, funding agencies should prioritize investment in next-generation spatial imaging platforms—such as nano-DESI, AFM-IR, and 3D multimodal systems—to resolve subcellular metabolic detail. Second, stronger academia–industry partnerships are needed to accelerate the development and implementation of scalable, AI-powered analysis pipelines. Third, establishing international spatial metabolomics consortia and open-access data repositories will foster reproducibility, enable cross-cohort validation, and drive translational biomarker discovery.

In summary, spatial metabolomics is poised to transform cancer research and clinical practice. Realizing this potential will require coordinated efforts in technology development, interdisciplinary collaboration, and infrastructure expansion to bridge discovery and application in precision oncology.

## Data Availability

The original contributions presented in the study are included in the article/**Supplementary Material**. Further inquiries can be directed to the corresponding author/s.

## References

[1] Bray F Laversanne M Sung H Ferlay J Siegel RL Soerjomataram I . Global cancer statistics 2022: GLOBOCAN estimates of incidence and mortality worldwide for 36 cancers in 185 countries. CA: Cancer J Clin. (2024) 74:229–63. doi: 10.3322/caac.21834 38572751

[2] van der Pol Y Mouliere F . Toward the early detection of cancer by decoding the epigenetic and environmental fingerprints of cell-free DNA. Cancer Cell. (2019) 36:350–68. doi: 10.1016/j.ccell.2019.09.003 31614115

[3] Bian W Zhang B Song Z Knisbacher BA Chan YM Bao C . SplitFusion enables ultrasensitive gene fusion detection and reveals fusion variant-associated tumor heterogeneity. Patterns (N Y). (2025) 6:101174. doi: 10.1016/j.patter.2025.101174 40041857 PMC11873004

[4] Chen S Liu J He G Tang N Zeng Y . Research hotspots and trends in global cancer immunometabolism:A bibliometric analysis from 2000 to 2023. J Multidiscip Healthc. (2024) 17:5117–37. doi: 10.2147/JMDH.S495330 PMC11568773 39553266

[5] Shen D Min J Chen J Yan D Han J Liu H . Study on the material basis and mechanisms of achyrocline satureioides in the treatment of nonsmall cell lung cancer based on network pharmacology and spatial metabolomics. Anal Chem. (2025) 97(10):5688–97. doi: 10.1021/acs.analchem.4c06682 40036484

[6] Li B Sun C Yang Y Li C Zheng T Zhou J . Spatial metabolomics revealed multi-organ toxicity and visualize metabolite changes induced by borneol in zebrafish. Sci Total Environ. (2025) 968:178886. doi: 10.1016/j.scitotenv.2025.178886 39986037

[7] Zeng H Chi H . mTOR signaling in the differentiation and function of regulatory and effector T cells. Curr Opin Immunol. (2017) 46:103–11. doi: 10.1016/j.coi.2017.04.005 PMC5554750 28535458

[8] Patsoukis N Bardhan K Chatterjee P Sari D Liu B Bell LN . PD-1 alters T-cell metabolic reprogramming by inhibiting glycolysis and promoting lipolysis and fatty acid oxidation. Nat Commun. (2015) 6:6692. doi: 10.1038/ncomms7692 25809635 PMC4389235

[9] Young LEA Conroy LR Clarke HA Hawkinson TR Bolton KE Sanders WC . *In situ* mass spectrometry imaging reveals heterogeneous glycogen stores in human normal and cancerous tissues. EMBO Mol Med. (2022) 14:e16029. doi: 10.15252/emmm.202216029 36059248 PMC9641418

[10] Wu Z Chen S Wang Y Li F Xu H Li M . Current perspectives and trend of computer-aided drug design: a review and bibliometric analysis. Int J Surg. (2024) 110:3848–78. doi: 10.1097/JS9.0000000000001289 PMC11175770 38502850

[11] Sun C Li T Song X Huang L Zang Q Xu J . Spatially resolved metabolomics to discover tumor-associated metabolic alterations. Proc Natl Acad Sci U.S.A. (2019) 116:52–7. doi: 10.1073/pnas.1808950116 PMC6320512 30559182

[12] Asp M Bergenstråhle J Lundeberg J . Spatially resolved transcriptomes-next generation tools for tissue exploration. Bioessays. (2020) 42:e1900221. doi: 10.1002/bies.201900221 32363691

[13] Liu Y Yang M Deng Y Su G Enninful A Guo CC . High-spatial-resolution multi-omics sequencing via deterministic barcoding in tissue. Cell. (2020) 183:1665–1681.e18. doi: 10.1016/j.cell.2020.10.026 33188776 PMC7736559

[14] Ma R-Y Black A Qian B-Z . Macrophage diversity in cancer revisited in the era of single-cell omics. Trends Immunol. (2022) 43:546–63. doi: 10.1016/j.it.2022.04.008 35690521

[15] Williams CG Lee HJ Asatsuma T Vento-Tormo R Haque A . An introduction to spatial transcriptomics for biomedical research. Genome Med. (2022) 14:68. doi: 10.1186/s13073-022-01075-1 35761361 PMC9238181

[16] Moffitt JR Lundberg E Heyn H . The emerging landscape of spatial profiling technologies. Nat Rev Genet. (2022) 23:741–59. doi: 10.1038/s41576-022-00515-3 35859028

[17] Xu C Jin X Wei S Wang P Luo M Xu Z . DeepST: identifying spatial domains in spatial transcriptomics by deep learning. Nucleic Acids Res. (2022) 50:e131. doi: 10.1093/nar/gkac901 36250636 PMC9825193

[18] Parrot D Papazian S Foil D Tasdemir D . Imaging the unimaginable: desorption electrospray ionization - imaging mass spectrometry (DESI-IMS) in natural product research. Planta Med. (2018) 84:584–93. doi: 10.1055/s-0044-100188 PMC6053038 29388184

[19] Planque M Igelmann S Ferreira Campos AM Fendt SM . Explainable multiview framework for dissecting spatial relationships from highly multiplexed data. Genome Biol. (2022) 23:97. doi: 10.1016/j.cbpa.2023.102362 35422018 PMC9011939

[20] Wu Y Cheng Y Wang X Fan J Gao Q . Spatial omics: Navigating to the golden era of cancer research. Clin Transl Med. (2022) 12:e696. doi: 10.1002/ctm2.696 35040595 PMC8764875

[21] Ma X Fernández FM . Advances in mass spectrometry imaging for spatial cancer metabolomics. Mass Spectrom Rev. (2024) 43:235–68. doi: 10.1002/mas.21804 PMC9986357 36065601

[22] Hsieh WC Budiarto BR Wang YF Lin CY Gwo MC So DK . Spatial multi-omics analyses of the tumor immune microenvironment. J BioMed Sci. (2022) 29:96. doi: 10.1186/s12929-022-00879-y 36376874 PMC9661775

[23] Zhu Z Cai J Hou W Xu K Wu X Song Y . Microbiome and spatially resolved metabolomics analysis reveal the anticancer role of gut Akkermansia muciniphila by crosstalk with intratumoral microbiota and reprogramming tumoral metabolism in mice. Gut Microbes. (2023) 15:2166700. doi: 10.1080/19490976.2023.2166700 36740846 PMC9904296

[24] Zang Q Sun C Chu X Li L Gan W Zhao Z . Spatially resolved metabolomics combined with multicellular tumor spheroids to discover cancer tissue relevant metabolic signatures. Anal Chim Acta. (2021) 1155:338342. doi: 10.1016/j.aca.2021.338342 33766316

[25] Mosquera MJ Kim S Bareja R Fang Z Cai S Pan H . Extracellular matrix in synthetic hydrogel-based prostate cancer organoids regulate therapeutic response to EZH2 and DRD2 inhibitors. Adv Mater. (2022) 34:e2100096. doi: 10.1002/adma.202100096 34676924 PMC8820841

[26] Hu T Allam M Cai S Henderson W Yueh B Garipcan A . Single-cell spatial metabolomics with cell-type specific protein profiling for tissue systems biology. Nat Commun. (2023) 14:8260. doi: 10.1038/s41467-023-43917-5 38086839 PMC10716522

[27] Neumann JM Freitag H Hartmann JS Niehaus K Galanis M Griesshammer M . Subtyping non-small cell lung cancer by histology-guided spatial metabolomics. J Cancer Res Clin Oncol. (2022) 148:351–60. doi: 10.1007/s00432-021-03834-w PMC8800912 34839410

[28] Planque M Igelmann S Ferreira Campos AM Fendt SM . Spatial metabolomics principles and application to cancer research. Curr Opin Chem Biol. (2023) 76:102362. doi: 10.1016/j.cbpa.2023.102362 37413787

[29] Kundu M Butti R Panda VK Malhotra D Das S Mitra T . Modulation of the tumor microenvironment and mechanism of immunotherapy-based drug resistance in breast cancer. Mol Cancer. (2024) 23:92. doi: 10.1186/s12943-024-01990-4 38715072 PMC11075356

[30] Marconato L Palla G Yamauchi KA Virshup I Heidari E Treis T . SpatialData: an open and universal data framework for spatial omics. Nat Methods. (2025) 22:58–62. doi: 10.1038/s41592-024-02212-x 38509327 PMC11725494

[31] Dhanasekaran R Suzuki H Lemaitre L Kubota N Hoshida Y. . Molecular and immune landscape of hepatocellular carcinoma to guide therapeutic decision-making. Hepatology. (2025) 81:1038–57. doi: 10.1097/hep.0000000000000513 PMC10713867 37300379

[32] Wang Q Sun N Meixner R Le Gleut R Kunzke T Feuchtinger A . Metabolic heterogeneity in adrenocortical carcinoma impacts patient outcomes. JCI Insight. (2023) 8:e167007. doi: 10.1172/jci.insight.167007 37606037 PMC10543722

[33] Chen G Li Y Geng S Lv L Wang Y Li X . Evaluating the heterogeneity of advanced prostate cancer by 18F-DCFPyL and 18F-FDG PET/CT in a prospective cohort. Prostate. (2025) 85(8):749–57. doi: 10.1002/pros.24881 40045414

[34] Zhu Z Zhang Y Wang L Geng H Li M Chen S . Spatial metabolomics profiling reveals curcumin induces metabolic reprogramming in three-dimensional tumor spheroids. Metabolites. (2024) 14:482. doi: 10.3390/metabo14090482 39330489 PMC11433860

[35] Sun N Krauss T Seeliger C Kunzke T Stöckl B Feuchtinger A . Inter-organ cross-talk in human cancer cachexia revealed by spatial metabolomics. Metabolism. (2024) 161:156034. doi: 10.1016/j.metabol.2024.156034 39299512

[36] Zou W Huo B Tu Y Zhu Y Hu Y Li Q . Metabolic reprogramming by chemo-gene co-delivery nanoparticles for chemo-immunotherapy in head and neck squamous cell carcinoma. Acta Biomater. (2025) S1742-7061(25):00272–7. doi: 10.1016/j.actbio.2025.04.031 40252747

[37] Wu JY Huang TW Hsieh YT Wang YF Yen CC Lee GL . Cancer-derived succinate promotes macrophage polarization and cancer metastasis via succinate receptor[J. Mol Cell. (2020) 77:213–27. doi: 10.1016/j.molcel.2019.10.023 31735641

[38] Ma X Bi E Lu Y Su P Huang C Liu L . Cholesterol induces CD8(+) T cell exhaustion in the tumor microenvironment. Cell Metab. (2019) 30:143–56. doi: 10.1016/j.cmet.2019.04.002 PMC7061417 31031094

[39] Sun C Wang A Zhou Y Chen P Wang X Huang J . Spatially resolved multi-omics highlights cell-specific metabolic remodeling and interactions in gastric cancer. Nat Commun. (2023) 14:2692. doi: 10.1038/s41467-023-38360-5 37164975 PMC10172194

[40] Liang W Zhu Z Xu D Wang P Guo F Xiao H . The burgeoning spatial multi-omics in human gastrointestinal cancers. PeerJ. (2024) 12:e17860. doi: 10.7717/peerj.17860 39285924 PMC11404479

[41] Lu Y Han X Zhang H Zheng L Li X . Multi-omics study on the molecular mechanism of anlotinib in regulating tumor metabolism. Eur J Pharmacol. (2024) 975:176639. doi: 10.1016/j.ejphar.2024.176639 38729415

[42] Khanijou JK Kulyk H Bergès C Khoo LW Ng P Yeo HC . Metabolomics and modelling approaches for systems metabolic engineering. Metab Eng Commun. (2022) 15:e00209. doi: 10.1016/j.mec.2022.e00209 36281261 PMC9587336

[43] Shen J Sun N Zens P Kunzke T Buck A Prade VM . Spatial metabolomics for evaluating response to neoadjuvant therapy in non-small cell lung cancer patients. Cancer Commun (Lond). (2022) 42:517–35. doi: 10.1002/cac2.12310 PMC9198346 35593195

[44] Zhi Y Wang Q Zi M Zhang S Ge J Liu K . Spatial transcriptomic and metabolomic landscapes of oral submucous fibrosis-derived oral squamous cell carcinoma and its tumor microenvironment. Adv Sci (Weinh). (2024) 11:e2306515. doi: 10.1002/advs.202306515 38229179 PMC10966560

[45] Zhang Z Bao C Jiang L Wang S Wang K Lu C . When cancer drug resistance meets metabolomics (bulk, single-cell and/or spatial): Progress, potential, and perspective. Front Oncol. (2022) 12:1054233. doi: 10.3389/fonc.2022.1054233 36686803 PMC9854130

